# Increased Invasive Pneumococcal Disease, North East England, UK

**DOI:** 10.3201/eid2301.160897

**Published:** 2017-01

**Authors:** Catherine Houseman, Gareth J. Hughes, Kaye E. Chapman, Deborah Wilson, Russell Gorton

**Affiliations:** Public Health England, Newcastle upon Tyne, UK (C. Houseman, K.E. Chapman, D. Wilson, R. Gorton);; Public Health England, Leeds, UK (G.J. Hughes)

**Keywords:** Streptococcus pneumoniae, pneumococcal infections, invasive pneumococcal disease, pneumococcal polysaccharide vaccine, PCV13 vaccine, incidence, bacteria, England, vaccines, United Kingdom

## Abstract

Since April 2014, invasive pneumococcal disease incidence has increased substantially across North East England, United Kingdom, reversing the decline that followed the 2006 introduction of pneumococcal conjugate vaccines. Significant increases occurred in 23-valent polysaccharide vaccine serotypes and nonvaccine serotypes. Trends in other regions and long-term effects of multivalent vaccines require further investigation.

The UK routine immunization program includes 2 vaccines against pneumococcal disease (*1*). The 7-valent pneumococcal conjugate vaccine (PCV7), introduced in 2006 and replaced by the 13-valent pneumococcal conjugate vaccine (PCV13) in 2010, is given to infants 2, 4, and 12 months of age (*1*). The 23-valent pneumococcal polysaccharide vaccine (PPV23) has been recommended for persons in clinically defined risk groups >2 years of age since 1992 and for all persons >65 years of age since 2003 (*1*). National coverage of PCV at 12 months reached 90% by epidemiologic year (April 1–March 31, indicated by slashes in year ranges) 2008/2009 and remains >93% (*2*). Since 2009/2010, coverage in North East England (NEE) has been >95% (*2*). By 2007/2008, PPV coverage in England and NEE reached 70% among all persons >65 years of age and remained there through March 31, 2016 (*3*).

Invasive pneumococcal disease (IPD) incidence in NEE declined significantly after the introduction of PCV7 and subsequently PCV13 among persons in vaccinated and nonvaccinated age groups, consistent with other countries and the United Kingdom (*4**–**8*). This decline coincided with emergence of less frequent nonvaccine type (NVT) serotypes, reinforcing the need for continued IPD surveillance (*4**–**8*). Using enhanced surveillance data for April 1, 2006, through March 31, 2016, we detected increased IPD incidence in NEE. 

## The Study 

In April 2006, the NEE Invasive Pneumococcal Disease Enhanced Surveillance System was established (*4*) and gathered data from microbiology services, hospitals and primary care clinicians, and the Public Health England Respiratory and Vaccine Preventable Bacteria Reference Unit (*9*). We compared IPD incidence during the 2015/2016 epidemiologic year with that from previous epidemiologic years and with the average annual incidence during the 3 epidemiologic years covering April 1, 2011–March 31, 2014. We analyzed IPD incidence across all cases combined and cases stratified by vaccine serotype subgroups: PCV7/PCV13 serotypes (1, 3, 4, 5, 6A, 6B, 7F, 9V, 14, 18C, 19A, 19F, 23F); PPV23-exclusive serotypes (2, 8, 9N, 10A, 11A, 12F, 15B [including 15B/C], 17F, 20, 22F, 33F); and NVT serotypes (*1*). We examined IPD incidence trends by specific serotype during April 2013–March 2016 by using incidence rate ratios (IRRs), estimated by using negative binomial regression (with counts per calendar quarter, robust standard errors, and offset with the natural logarithm of the NEE population [*10*]).

For each epidemiologic year spanning April 1, 2011–March 31, 2014, an average of 211 IPD cases (8.1 cases/100,000 population) were reported. In contrast, during 2015/2016, a total of 298 cases (11.4/100,000) were reported. This incidence was significantly greater than that for 2014/2015 (230 cases, 8.8/100,000; IRR 1.30, 95% CI 1.09–1.55, p = 0.003); significantly greater than the average during the 3 epidemiologic years spanning 2011–2014 (IRR 1.40, 95% CI 1.17–1.68, p<0.001); and similar to 2006/2007 (11.91/100,000; IRR 0.96, 95% CI 0.81–1.12, p = 0.577) (Table 1). A similar trend occurred among patients 5–64 years of age (2015/2016 vs. 2014/2015 IRR 1.32, 95% CI 1.01–1.73, p = 0.036; 2015/2016 vs. 2011–2014 IRR 1.43, 95% CI 1.09–1.88, p = 0.008; 2015/2016 vs. 2006/2007 IRR 0.97, 95% CI 0.76–1.24, p = 0.796) and patients >65 years of age (2015/2016 vs. 2014/2015 IRR 1.25, 95% CI 0.98–1.61, p = 0.067; 2015/2016 vs. 2011–2014 IRR 1.40, 95% CI 1.08–1.82, p = 0.008; 2015/2016 vs. 2006/2007 IRR 0.98, 95% CI 0.77–1.25, p = 0.892) (Table 1). Among patients <5 years of age, incidence during 2015/2016 remained significantly lower than that during 2006/2007 (IRR 0.44, 95% CI 0.23–0.80, p = 0.004), similar to that during 2011–2014 (IRR 0.99, 95% CI 0.48–2.07, p = 0.985), and did not significantly increase during 2014/2015 (IRR 1.55, 95% CI 0.68–3.65, p = 0.265) (Table 1).

**Table 1:** Number and incidence of invasive pneumococcal disease in North East England, April 2006–March 2016

Epidemiologic year*	Total no. cases	No. (%) cases, by age group†				Incidence rate (95% CI)‡			
		<5 y	5–64 y	≥65 y		All cases	<5 y	5–64 y	>65 y
2006/2007	304	35 (12)	137 (45)	132 (43)		11.91 (10.71–13.22)	25.56 (18.99–33.46)	6.89 (5.86–8.09)	30.82 (26.63–35.34)
2007/2008	268	29 (11)	129 (48)	110 (41)		10.46 (9.33–11.71)	20.67 (14.79–28.1)	6.48 (5.48–7.64)	25.57 (21.68–29.9)
2008/2009	263	32 (12)	119 (45)	112 (43)		10.24 (9.12–11.47)	22.21 (16.19–29.67)	5.97 (5.02–7.1)	25.85 (21.95–30.17)
2009/2010	251	27 (11)	114 (45)	110 (44)		9.75 (8.66–10.95)	18.48 (13.03–25.56)	5.73 (4.79–6.83)	25.1 (21.26–29.36)
2010/2011	263	29 (11)	117 (44)	117 (44)		10.17 (9.06–11.39)	19.53 (13.95–26.64)	5.87 (4.92–6.99)	26.27 (22.39–30.54)
2011/2012	237	21 (9)	98 (41)	118 (50)		9.13 (8.08–10.30)	14 (9.34–20.46)	4.92 (4.05–5.95)	26.07 (22.24–30.31)
2012/2013	226	18 (8)	107 (47)	101 (45)		8.68 (7.66–9.83)	11.86 (7.63–17.96)	5.4 (4.49–6.48)	21.55 (18.07–25.5)
2013/2014	169	12 (7)	74 (44)	83 (49)		6.47 (5.59–7.48)	7.91 (4.58–13.31)	3.74 (2.99–4.67)	17.28 (14.17–20.92)
2014/2015	230	11 (5)	100 (43)	119 (52)		8.78 (7.76–9.93)	7.26 (4.1–12.52)	5.06 (4.18–6.12)	24.21 (20.63–28.19)
2015/2016	298	17 (6)	132 (44)	149 (50)		11.38 (10.22–12.65)	11.21 (7.12–17.22)	6.68 (5.66–7.87)	30.32 (26.42–34.52)

*April 1–March 31.  †Percentage of all cases.  ‡No. cases/100,000 population. Mid-year population estimates were obtained from the Office for National Statistics (*10*); for all epidemiologic years other than 2015/2016, incidence rates were calculated by using mid-year population estimates for the first calendar year; for 2015/2016, mid-year estimates for 2014 were used.

**Figure 1:**
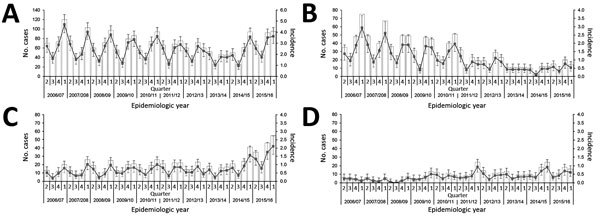
Number and incidence (no. cases/100,000 population) of invasive pneumococcal disease cases by vaccine type serotype subgroups in North East England, by quarter April 2006–March 2016. A) All cases. B) Cases caused by 13-valent pneumococcal conjugate vaccine serotypes. C) Cases caused by 23-valent pneumococcal polysaccharide vaccine serotypes, excluding those also contained in PCV13. D) Cases caused by nonvaccine types. Bars show numbers of cases. Lines indicate incidence: error bars indicate 95% CIs.

**Figure 2:**
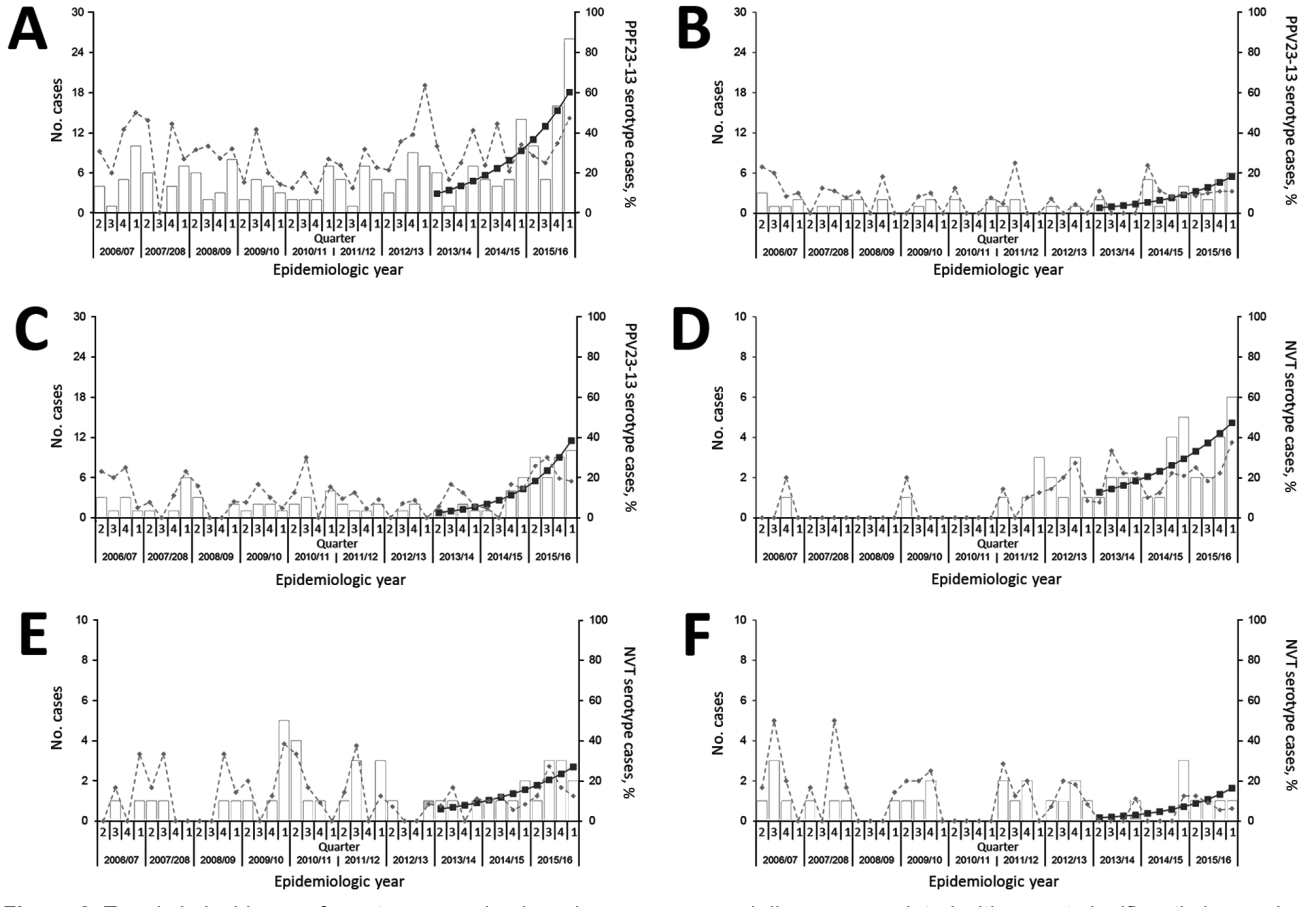
Trends in incidence of serotypes causing invasive pneumococcal disease associated with recent significantly increasing incidence in North East England, by quarter, April 2006–March 2016. Panels show trends by individual serotypes: A) serotype 8; B) serotype 9N; C) serotype 12F; D) serotype 15A; E) serotype 23F; F) serotype 35F. Bars show observed numbers of cases; broken lines show the percentage of all serotype group cases (A–C PPV23–13; D–F NVT); solid lines show counts of cases predicted by a negative binomial regression model for April 2013–March 2016. NVT, nonvaccine type serotype cases; PPV23-13, 23–valent pneumococcal polysaccharide vaccine serotype cases excluding those also contained in the 13–valent pneumococcal conjugate vaccine.

The recent rise in IPD is largely attributable to increased cases caused by PPV23-exclusive serotypes (2015/2016 vs. 2011–2014 IRR 2.42, 95% CI 1.80–3.29, p<0.001; 2015/2016 vs. 2006/2007 IRR 3.04, 95% CI 2.20–4.27, p<0.001), notable from 2014/2015 on (Figure 1). Of the 11 serotypes exclusive to PPV23, significant increasing trends were demonstrated by serotypes 8, 9N, and 12F from 2013/2014 on (Table 2; Figure 2). This trend was observed among patients 5–64 and >65 years of age; cases among patients <5 years of age were considerably fewer, and temporal changes by serotype were difficult to interpret (data not shown).

**Table 2:** Trends in incidence of serotypes causing invasive pneumococcal disease in North East England, April 2013–March 2016*

Serotype group, serotype	IRR (95% CI)†	p value†
PPV23–13		
** 8**	**1.18 (1.08–1.29)**	**<0.001**
** 9N**	**1.19 (1.04–1.36)**	**0.009**
10A	1.07 (0.89–1.29)	0.465
11A	1.03 (0.92–1.15)	0.617
** 12F**	**1.28 (1.20–1.36)**	**<0.001**
15B/C‡	1.29 (0.99–1.69)	0.061
17F	0.84 (0.65–1.09)	0.188
20	1.12 (0.96–1.31)	0.148
22F	1.04 (0.95–1.13)	0.421
33F	1.17 (1.00–1.38)	0.051
NVT§		
6C	1.00 (0.91–1.10)	0.958
** 15A**	**1.13 (1.07–1.19)**	**<0.001**
16F	0.99 (0.85–1.15)	0.924
** 23A**	**1.15 (1.06–1.24)**	**<0.001**
23B	1.06 (0.81–1.40)	0.657
24F	1.03 (0.91–1.17)	0.645
31	1.03 (0.92–1.16)	0.591
35B	1.12 (0.92–1.37)	0.256
** 35F**	**1.23 (1.06–1.44)**	**0.008**
38	0.84 (0.61–1.15)	0.279

*Boldface indicates significance. IRR, incidence rate ratio; PPV23–13, 23–valent pneumococcal polysaccharide vaccine serotype cases, excluding those also contained in the 13–valent pneumococcal conjugate vaccine; NVT, nonvaccine type serotype cases.  †Increase in IRR per calendar quarter, estimated by using negative binomial regression, with counts per calendar quarter (from April 1–June 30, 2013 (Quarter 2) to January 1–March 31, 2016 (Quarter 1), robust standard errors and offset with the natural logarithm of the North East England population (*10*).  ‡Includes 15B and 15B/C but excludes 15C serotypes as determined by the Public Health England Respiratory and Vaccine Preventable Bacteria Reference Unit.  §NVT serotypes reported more than once in the epidemiologic year 2015–2016.

Over the longer term, the number of cases caused by NVT serotypes increased between 2006/2007 and 2015/2016 (IRR 2.58, 95% CI 1.52–4.56, p<0.001), particularly from 2008/2009 on (Figure 1). The increased incidence of IPD caused by NVT was not statistically significant between 2015/2016 and 2011–2014 (IRR 1.23, 95% CI 0.80–1.88, p = 0.236). Among NVTs with an observed increase, serotypes 15A, 23A, and 35F increased significantly from 2013/2014 on (Table 2; Figure 2). For 23A, this increase was particularly notable among persons >65 years of age; for serotypes 15A and 35F, the increase was among persons 5–64 and >65 years of age (data not shown).

## Conclusions

Total IPD incidence increased significantly, starting in 2014/2015, reversing the declines in total IPD incidence that followed the introduction of PCVs (*4**–**8*). The increases were significant for PPV23-exclusive serotypes 8, 9N, and 12F and for NVT serotypes 15A, 23A, and 35F, most notably among persons 5–64 and >65 years of age.

We know of no mechanism for increased host susceptibility that could explain these rapid incidence changes. Although associations between influenza and IPD have been reported (*11*,*12*) and genetically drifted influenza strains contributed to low vaccine effectiveness in the United Kingdom during 2014/2015 (*13*), our primary analysis compared 2015/2016 with 2011–2014 so that any IPD increase associated with the 2014–2015 influenza season had no influence on these findings.

Mechanisms for changes in serotype prevalence include serotype replacement and capsular switching (genetic serotype switch in individual organisms) (*14*). In NEE, serotype replacement and declining IPD incidence were observed among persons of all age groups soon after introduction of PCV7 childhood vaccination (*4*), highlighting the influence of strains affecting young children in determining prevalent pneumococcal serotypes among persons in nonvaccine age groups. With ongoing >95% vaccination coverage in NEE, direct protection extends into an ever-increasing proportion of the population, up to those 10 years of age in 2016, increasing pressure on PCV strains. This pressure may be leading to accelerated serotype replacement throughout the population or to increased capsular switching, resulting in some non-PCV serotypes becoming more prevalent. Natural fluctuations in serotype prevalence may also be occurring. However, explanations for the recent IPD increase need to account for the recent and somewhat sudden rise following a long period of decline. For instance, perhaps natural expansion of non-PCV strains into the ecologic niches created has been delayed and therefore the decline observed was only temporary, or perhaps there have been recent changes in invasiveness of the non-PCV strains either naturally or associated with serotype replacement or capsular switching. 

Our findings, together with data from all England (*15*), suggest that IPD epidemiology continues to evolve after 10 years of routine childhood vaccination. Observations from other regions that have introduced PCV are merited to determine whether the increase observed in NEE is, or becomes, a widespread phenomenon and, if so, its relationship to the timing of PCV implementation and PCV coverage. Also needed are further studies of the effects of ongoing vaccination on carriage and molecular studies to identify evidence for capsular switching and changes in invasiveness. Clarification of such factors may help guide changes to public health strategies required to tackle persistent IPD.
